# SIRGs score may be a predictor of prognosis and immunotherapy response for esophagogastric junction adenocarcinoma

**DOI:** 10.3389/fimmu.2022.977894

**Published:** 2022-08-16

**Authors:** Li-Ying OuYang, Zi-Jian Deng, Yu-Feng You, Jia-Ming Fang, Xi-Jie Chen, Jun-Jie Liu, Xian-Zhe Li, Lei Lian, Shi Chen

**Affiliations:** ^1^ Department of Intensive Care Unit, Sun Yat-Sen University Cancer Center, Guangzhou, China; ^2^ Department of Gastrointestinal Surgery, The Sixth Affiliated Hospital, Sun Yat-Sen University, Guangzhou, China; ^3^ Guangdong Institute of Gastroenterology, Guangzhou, China; ^4^ Guangdong Provincial Key Laboratory of Colorectal and Pelvic Floor Diseases, Guangzhou, China; ^5^ School of Medicine, Sun Yat-Sen University, Guangzhou, China

**Keywords:** esophagogastric junction adenocarcinoma, SIRGs score, prognosis, immunotherapy, tumor microenvironment

## Abstract

**Background:**

Esophagogastric junction adenocarcinoma (EGJA) is a special malignant tumor with unknown biological behavior. PD-1 checkpoint inhibitors have been recommended as first-line treatment for advanced EGJA patients. However, the biomarkers for predicting immunotherapy response remain controversial.

**Methods:**

We identified stromal immune-related genes (SIRGs) by ESTIMATE from the TCGA-EGJA dataset and constructed a signature score. In addition, survival analysis was performed in both the TCGA cohort and GEO cohort. Subsequently, we explored the differences in tumor-infiltrating immune cells, immune subtypes, immune-related functions, tumor mutation burden (TMB), immune checkpoint gene expression, immunophenoscore (IPS) between the high SIRGs score and low SIRGs score groups. Finally, two validation cohorts of patients who had accepted immunotherapy was used to verify the value of SIRGs score in predicting immunotherapy response.

**Results:**

Eight of the SIRGs were selected by LASSO regression to construct a signature score (SIRGs score). Univariate and multivariate analyses in the TCGA and GEO cohort suggested that SIRGs score was an independent risk factor for the overall survival (OS) and it could increase the accuracy of clinical prediction models for survival. However, in the high SIRGs score group, patients had more immune cell infiltration, more active immune-related functions, higher immune checkpoint gene expression and higher IPS-PD1 and IPS-PD1-CTLA4 scores, which indicate a better response to immunotherapy. The external validation illustrated that high SIRGs score was significantly associated with immunotherapy response and immune checkpoint inhibitors (ICIs) can improve OS in patients with high SIRGs score.

**Conclusion:**

The SIRGs score may be a predictor of the prognosis and immune-therapy response for esophagogastric junction adenocarcinoma.

## Introduction

Esophagogastric junction carcinoma is a kind of malignant tumor with a special location and unknown biological behaviors (1). Compared with distal gastric cancer (GC), esophagogastric junctional adenocarcinoma (EGJA) has lower differentiation and higher malignancy (2, 3). Unfortunately, most EGJA patients in China are in an advanced stage when diagnosed, with poor chemosensitivity and poor prognosis, with a 5-year survival rate of 14% ~ 22% (4). Therefore, it is very important to explore new treatment methods other than surgery, chemotherapy and radiotherapy for EGJA.

Immunotherapy is widely used in digestive tract malignancies, especially gastric cancer and esophageal cancer (5–7). However, at present, there are obvious differences in the understanding of this tumor between Europe, America and East Asia (8). In clinical studies in Europe, EGJA is often classified as esophageal cancer (9, 10), while in Asia, it is classified as gastric cancer (11). Although several biomarkers have been shown to predict the efficacy of the PD-1 inhibitor, none of them have been accurate enough (12). As a new therapeutic strategy, treatment aimed at the tumor microenvironment (TME) has attracted public attention (13). The TME is composed of a variety of cell types, including the matrix, blood vessels, secretory factors, surrounding matrix and the internal environment of tumor cells. It plays an important role in the occurrence, development and invasion of tumors (14, 15). As the TME is mainly determined by the genomic landscape of tumors (16), some algorithms, such as Estimation of Stromal and Immune cells in Malignant Tumor tissues using Expression data (ESTIMATE) and Tumor IMmune Estimation Resource (TIMER) methods (17, 18), have been developed to predict tumor purity and estimate the abundance of tumor-infiltrating immune cells based on the gene expression profile. Many studies have applied these big-data-based algorithms to various tumors, including cutaneous melanoma (19), prostate cancer (20), glioblastoma (21), and breast cancer (22), and validated their effectiveness; however, their utility in EGJA has not been investigated.

In our study, we employed the ESTIMATE algorithm to handle the RNA dataset downloaded from the TCGA database. We calculated the immune and stromal scores to identify the SIRGs to construct a signature for predicting the immunotherapy efficacy in EGJA.

## Materials and methods

### Gene expression datasets

We downloaded the transcriptome expression profiles and the clinicopathological data from the TCGA database. We calculated immune and stromal scores for each sample. Validation data were downloaded from the GEO database, including GSE66229 and GSE84437. Both of these groups of patients had the following clinicopathological characteristics: sex, age, tumor staging, etc.

### Differential expression analysis

We divided the patients into a high/low immune score group and a high/low stromal score group, which were evaluated by the ESTIMATE algorithm. Then, we identified differentially expressed genes (DEGs) by the “limma” package of R (4.1.0) in different immunoscore groups. A false discovery rate (FDR) <0.05 and a |log2-fold change |> 1 were screening criteria. The stromal-related DEGs were confirmed by the same methods. The genes that were co-upregulated/downregulated by the immune group and stromal group were selected as stromal-immune related genes (SIRGs).

### Pathway and function enrichment analysis

We used R software to explore the specific molecular mechanisms through Gene Ontology (GO) and Kyoto Encyclopedia of Genes and Genomes (KEGG) enrichment analyses for SIRGs using the “clusterProfiler” package.

### Survival analysis and construction of the SIRGs prognostic signature and SIRGs score-based nomogram

We used univariate Cox regression analysis to identify prognostic SIRGs. The SIRGs with p<0.05 were included in least absolute shrinkage and selection operator (LASSO) analysis to avoid overfitting (glmnet package). After screening by LASSO analysis, 8 selected IRGSs were used to construct a signature: SIRGs score = level of gene a * coefficient a + level of gene b * coefficient b + level of gene c * coefficient c + …… + level of gene n * coefficient. All EGJA patients were classified into high SIRGs group and low SIRGs group according to median SIRGs scores. Kaplan-Meier analysis and multivariate Cox regression were conducted to evaluate the efficiency of the SIRGs score in predicting prognosis (survival package).

In addition, SIRGs scores and clinical characteristics were included to construct a nomogram using the “RMS” package. Discrimination was verified by the Harrell concordance index (C-index) and area under the ROC curves (AUCs).

### Gene set enrichment analysis (GSEA) and single-sample GSEA (ssGSEA)

GSEA was carried out in high and low SIRGs groups by the package “org.Hs.eg.db” (23). To compare the state of immune function between high and low SIRGs group’s patients, ssGSEA was used to evaluate the 29 immune signature gene sets in each EGJA patient by the package “GSVA” (24, 25).

### TME-associated analysis

We calculated 22 types of infiltrating immune cells, including B cells, CD4+ T cells, CD8+ T cells, neutrophils, macrophages, and dendritic cells and so on, by using the R script from CIBERSORT. Then, we divided these 110 EGJA patients into 4 immune subtypes according to the characteristics of immune cell infiltration by unsupervised clustering.

### Tumor mutation burden (TMB) analysis

The mutation data was downloaded from TCGA (https://portal.gdc.cancer.gov/). The TMB score for each patient was calculated and analyzed using the “maftools” package. We exclude 3 patients without mutation data before TMB analysis.

### Predicting patient response to immunotherapy

We compared the expression of immune checkpoints and their ligands in different SIRGs score groups. The immunophenoscore (IPS) was obtained without bias by analyzing the expression of four categories of immunogenicity-determining genes: effector cells, immunosuppressive cells, MHC molecules, and immunomodulators. IPS was calculated on a range of 0–10 according to z scores representing gene expression in cell types. IPS was positively associated with the immunotherapeutic response. We downloaded the IPS for EGJA patients from the Cancer Immunome Atlas (TCIA, https://tcia.at/home).

### Statistical analysis

Clinicopathological factors associated with prognosis were determined by univariate and multivariate Cox regression. Kaplan–Meier survival curves were drawn by the package “survminer”, and differences in survival between the two groups were determined using the log-rank test. Statistical significance was set at two-sided p<0.05. Data were analyzed using SPSS v.22.0 (SPSS, Inc., Chicago, IL, USA) and R version 4.1.0.

## Results

### Identification of SIRGs by immunoscore and stromalscore

**Figure 1:**
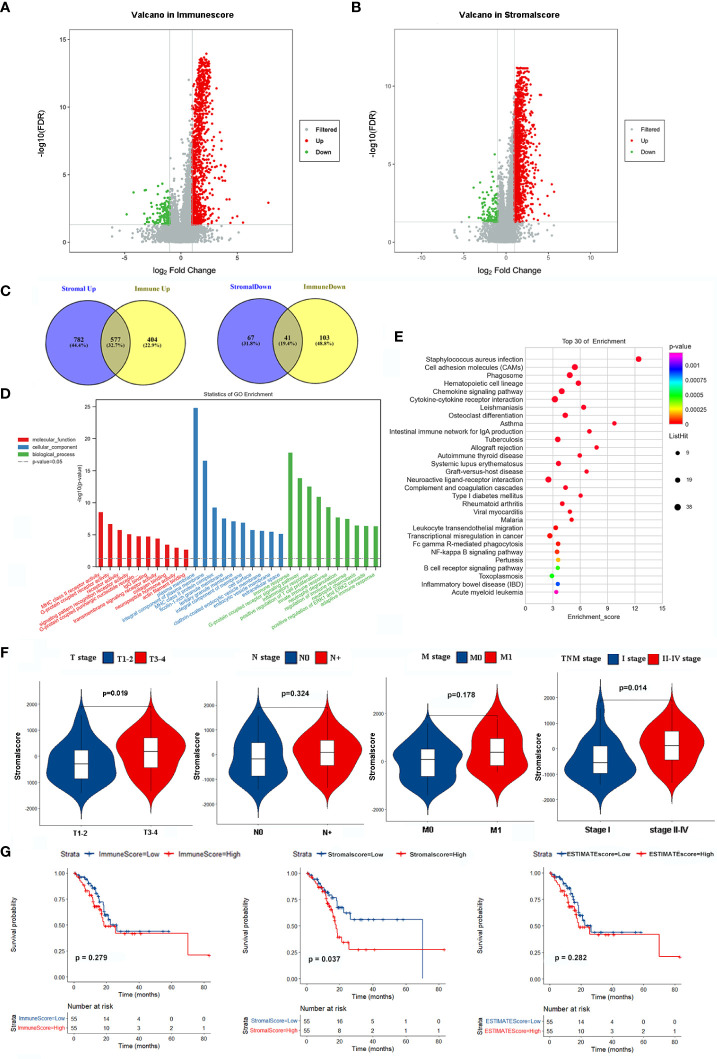
Identification SIRGs and enrichment analyses. **(A)** Volcano plot of DEGs in immunescore; **(B)** Volcano plot of DEGs in stromalscore; **(C)** Venn plot to identify SIRGs; **(D)** GO enrichment analysis; **(E)** KEGG pathway enrichment analysis; **(F)** The relationship between stromalscore and TNM stage; **(G)** Kaplan-Meier analysis in different groups.

The clinicopathological characteristics of 110 EGJA patients from the TCGA database are shown in **Table 1**. The ESTIMATE algorithm is applied for inferring the infiltration of immune cells and stromal cells in the microenvironment, and the results were revealed by immunescore and stromalscore. We separated the patients into the high-immunoscore group and the low-immunoscore group based on the median immunoscore. For comparison, there were 981 upregulated genes and 144 downregulated genes in the high immunescore EGJA patients (**Figure 1A**). Additionally, we divided these patients into high-stromalscore and low-stromalscore groups according to the median stromalscores. There were 1359 upregulated genes and 108 downregulated genes in the high-stromalscore EGJA patients (**Figure 1B**). As shown in the Venn diagram (**Figure 1C**), we defined overlapping genes that were up- or downregulated in the stromal and immune groups as stromal-immune related genes (SIRGs). We further used GO and KEGG pathway enrichment to analyze these SIRGs. The results demonstrated that immune response, plasma membrane, MHC class II receptor activity and another immune-related gene ontology were enriched (**Figure 1D, E**). The tumor-related stromal cell may participate in tumor progression, metastasis and chemotherapy response to further influence prognosis (26). We also found that the stromalscore was correlated with T stage and TNM stage, and EGJA patients in the high stromalscore group had higher T stage and TNM stage (**Figure 1F**). Survival analysis showed that EGJA patients with high stromalscore had a poorer prognosis than those with low stromalscore (p=0.037), which are consistent with the results of other researches (27–29). However, there was no significant survival difference between high immunescore patients and low immunoscore patients (**Figure 1G**, p=0.279).

**Table 1:** Clinical characteristics of EGJA and GC patients.

Variables	TCGA-EJGA cohort(n=110)	GEO-GC cohort(n=733)
Age (mean ± SD, years)	64.9 ± 10.9	60.8 ± 11.5
Gender		
Male	72(65.5%)	495(67.5%)
Female	38(34.5%)	238(32.5%)
T stage		
T1-T2	35(31.8%)	237(32.3%)
T3-T4	75(68.2%)	496(67.7%)
unknown	0(0%)	0(0%)
N stage		
N0	32(29.1%)	118(16.1%)
N+	78(70.9%)	615(83.9%)
unknown	0(0%)	0(0%)
M stage		
M0	102(92.7%)	273(26.4%)
M1	8(7.3%)	27(2.6%)
unknown		433(100%)
Stage		
I	19(17.3%)	31(4.2%)
II	34(30.9%)	97(13.2%)
III	45(40.9%)	95(13.0%)
IV	12(10.9%)	77(10.5%)
unknown	0(0%)	433(59.1%)
SIRGs score (mean ± SD)	0.34 ± 0.41	0.27 ± 0.16

### Survival analysis of SIRGs and gene set enrichment analysis (GSEA)

**Figure 2:**
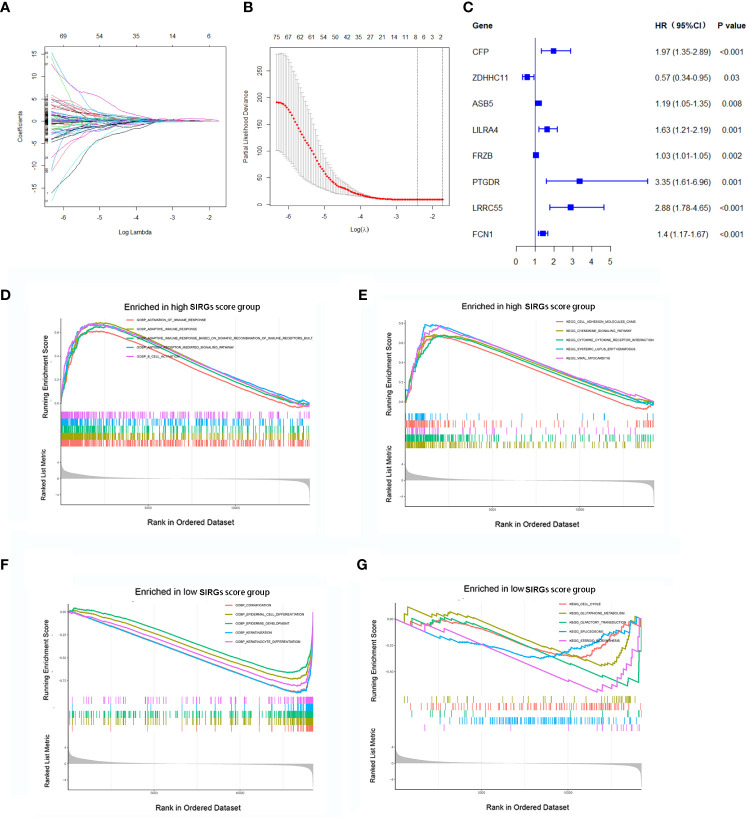
Construction of SIRGs score by LASSO analysis. **(A, B)** The LASSO Cox analysis identified that eight core SIRGs were associated with the prognosis of EGJA patients; **(C)** Forest plot of hazard ratios for eight core prognostic SIRGs; **(D, E)** GSEA analysis in high SIRGs score group; **(F, G)** GSEA analysis in high SIRGs score group.

We further explored the prognostic value of these 617 SIRGs. Univariate Cox analysis showed that 122 of them were associated with the prognosis of the EGJA patients, as shown in **Supplementary S1**. To avoid overfitting, a further LASSO analysis identified that 8 of 122 genes were core prognostic factors for EGJA patients (**Figures 2A, B**). We further used multivariate Cox proportional hazards regression analysis to construct a predictive model: SIRGs score=(0.1582*CFP)+(-0.06340*ZDHHC11) +(0.05201*ASB5)+(0.09763*LILRA4)+(0.002203*FRZB)+(0.004185*PTGDR)+(0.5599*LRRC55)+(0.1443*FCN1). The contribution of 8 core SIRGs on the overall survival are shown in **Figure 2C**. CFP (p<0.001), ASB5 (p=0.008), LILRA4 (p=0.001), FRZB (p=0.002), PTGDR (p=0.001), LRRC55 (p<0.001) and FCN1 (p<0.001) were prognostic risk factors for the EGJA patients. Among them, only ZDHHCC11(p=0.030) was a prognostic protective factor. These 8 SIRGs of Kaplan–Meier survival curves are show in **Supplementary S2**.

Furthermore, GO- and KEGG-related GSEA in the high SIRGs score group revealed that activation of multiple immune responses was enriched in GO biological processes (GOBP) and that cell adhesion molecules, chemokine signaling pathways and cytokine–cytokine receptor interactions were enriched in KEGG pathways (**Figures 2D, E**). In the low SIRGs score group, the GSEA enrichment focused on cornification, epidermal cell differentiation and epidermal development in GOBP and cell cycle, glutathione metabolism and olfactory transduction in KEGG (**Figures 2F, G**).

**Figure 3:**
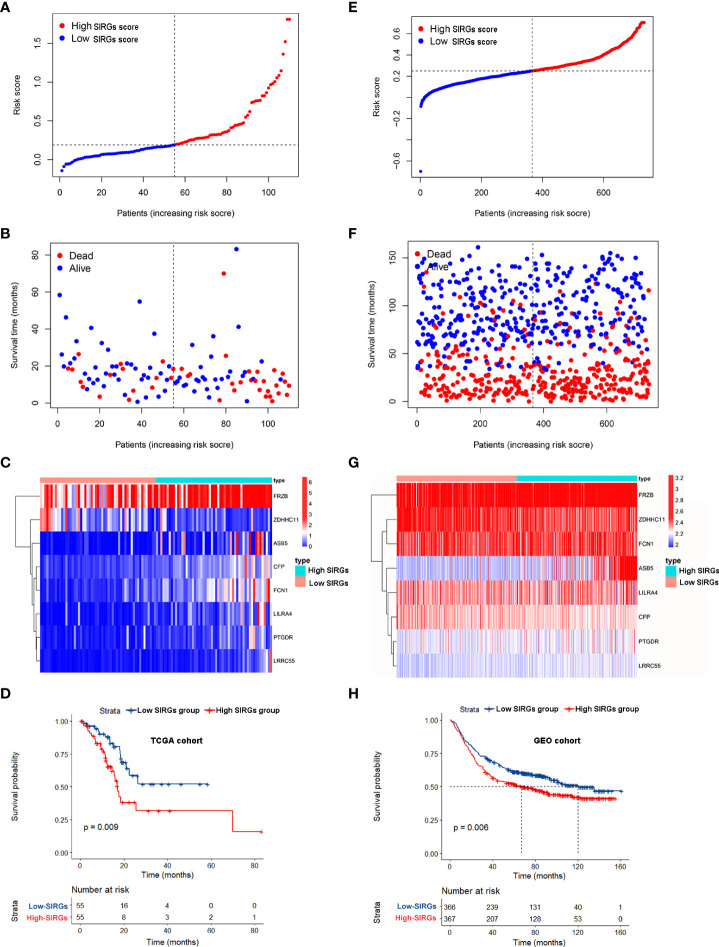
Survival analysis of SIRGs score in TCGA cohort and GEO cohort. **(A, E)** The rank of SIRGs scores; **(B, F)** The distribution of SIRGs score and overall survival time; **(C, G)** The heatmap of expression patterns of 8 SIRGs in low- and high-SIRGs score group; **(D, H)** Survival curves of different SIRGs score group.

We calculated the SIRGs score of the 110 EGJA patients by this formula and ranked the SIRGs score (**Figure 3A**). The dot plot in **Figure 3B** shows the distribution of SIRGs score and overall survival time. Then, we divided them into a high SIRGs score group and a low SIRGs score group according to the median value. The heatmap in **Figure 3C** illustrates the expression patterns of 8 SIRGs in the low and high SIRGs score groups. Kaplan–Meier analysis showed that the prognosis of the low SIRGs score group was better than that of the high SIRGs score group (**Figure 3D**, p=0.009).

We used the GEO gastric cancer database to validate this formula. A total of 733 gastric cancer patients from GSE66229 and GSE84437 were involved to calculate the SIRGs score. We also divided these patients into a GEO high SIRGs score group and a GEO low SIRGs score group. There were 367 and 366 patients in these two groups, respectively. We also ranked the SIRGs score, and the results are shown in **Figure 3E**. The distribution of SIRGs score and overall survival time were exhibited in **Figure 3F**. The expression patterns of 8 SIRGs showed as heatmap in **Figure 3G**. Kaplan–Meier analysis showed that the prognosis of the GEO low SIRGs score group was better than that of the GEO high SIRGs score group (**Figure 3H**, p=0.006). The median survival of these two groups was 67 months and 120 months, respectively.

### SIRGS-score-based nomogram model to predict the prognosis of EGJA patients

**Figure 4:**
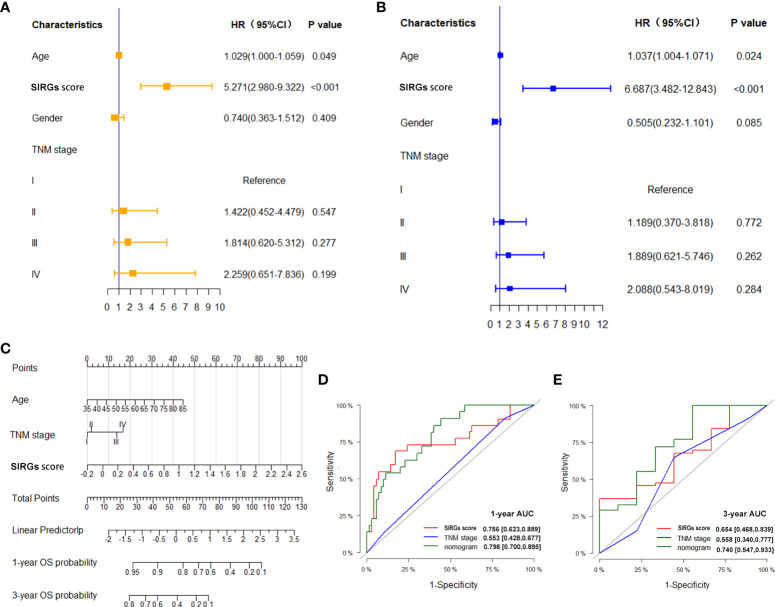
Establishment SIRGs score-based nomogram for predicting EGJA patients’ prognosis. **(A)** Forest plot presenting univariate Cox regression analysis result; **(B)** Forest plot presenting multivariate Cox regression analysis result; **(C)** SIRGs score-based nomogram; **(D)** AUC values of ROC predicted 1-year OS rates of Nomogram, SIRGs score and TNM stage; **(E)** AUC values of ROC predicted 3-year OS rates of Nomogram, SIRGs score and TNM stage.

Univariate and multivariate Cox analyses indicated that age and SIRGs score were independent prognostic factors for the TCGA-EGJA patients (**Figures 4A, B**) and GEO-GC patients (**Table 2**). However, TNM stage is widely considered a prognostic factor. Therefore, we also included TNM stage in the nomogram model (**Figure 4C**). The C-index of this model was 0.798. The AUCs of the 1-year and 3-year OS for the nomogram were 0.798 and 0.740, respectively. The prognostic test efficacy of the nomogram model containing the SIRGs score was better than that of the TNM staging (0.553 and 0.558) or the SIRGs score alone (0.756 and 0.654) (**Figures 4D, E**).

**Table 2:** Univariate and multivariate Cox regression analyses of overall survival for 733GC patients in the GEO cohort.

	Univariate Analysis		Multivariate Analysis	
	HR [95%CI]	P value	HR [95%CI]	P value
Age	1.016 [1.007 - 1.026]	0.001	1.018 [1.008 - 1.028]	<0.001
Sex		0.464		
male	Reference			
female	0.920 [0.735 - 1.151]			
T stage		0.023		0.079
T1-T2	Reference		Reference	
T3-T4	1.310 [1.038 - 1.653]		1.237 [0.975 - 1.570]	
N stage		0.004		0.004
N0	Reference		Reference	
N+	1.584 [1.162 - 2.159]		1.582 [1.160 - 2.157]	
SIRGs score	2.709 [1.464 - 5.010]	0.001	2.599 [1.386 - 4.872]	0.003

### Exploring the role of SIRGs in tumor immune cell infiltration, immune typing and immune function

**Figure 5:**
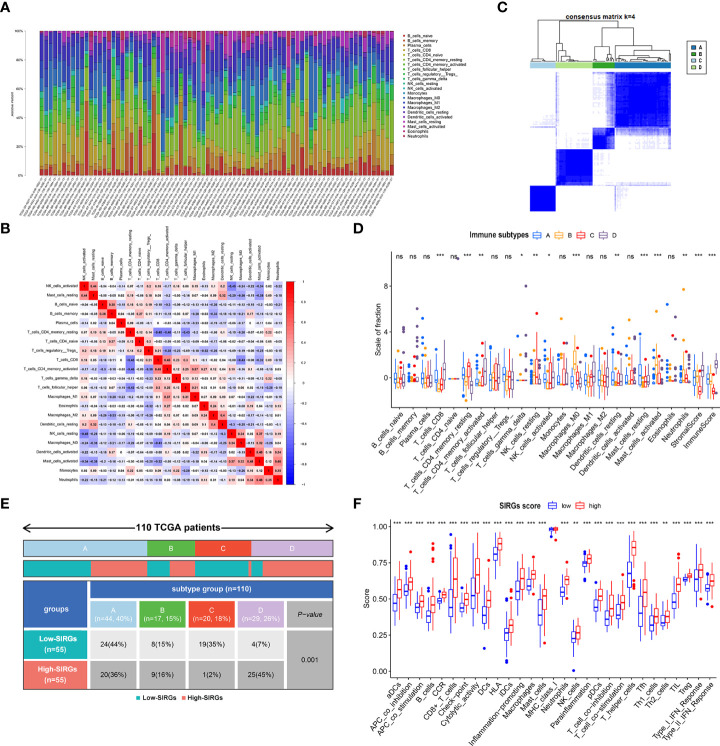
The difference TME in low- and high-SIRGs score group. **(A)** Relative proportion of immune infiltration in each EGJA patients; **(B)** The relationship in different immune infiltration cells; **(C)** Identify four immune subtypes by unsupervised clustering according to the immune infiltration state; **(D)** The difference infiltration immune cells in four immune subtypes; **(E)** The distribution of four immune subtypes in low- and high-SIRGs score group; **(F)** Immune-related functions analysis in in low- and high-SIRGs score group. *p<0.05, **p<0.01, ***p<0.001, ns, not significant.

The effect of immunotherapy for malignant tumors is often closely related to the tumor microenvironment. Tumor-infiltrating immune cells play essential roles in the TME. We further calculated the 22 types of tumor-infiltrating immune cells in 110 EGJA patients by CIBERSORT (30) (**Figure 5A**). The relationships of 22 types of infiltrating immune cells to each other are presented in the correlation matrix (**Figure 5B**). Then, these patients were divided into four immune types by unsupervised clustering algorithms according to infiltrating immune cells (**Figure 5C**). Moreover, in category D, we found that CD8+ T cells increased significantly, as did activated CD4+ T cells and NK cells (**Figure 5D**). Both stromal score and immunoscore were also significantly increased in category D (**Figure 5D**). We explored the distribution of these 4 categories in different SIRGs score groups. We found that in the high SIRGs score group, the proportion of type D accounted for 45%, which was much higher than that of the low SIRGs score group (**Figure 5E**, p=0.001). Subsequently, we explored the immune states between the high- and low SIRGs score groups by calculating the enrichment scores with ssGSEA. In total, 29 immune signature gene sets associated with immune status were analyzed. As **Figure 5F** shows, all 29 immune-state scores were higher in the high SIRGs score group, which suggested that those patients’ immune functions were more active.

### The relationship between SIRGs score and tumor mutation burden (TMB)

**Figure 6:**
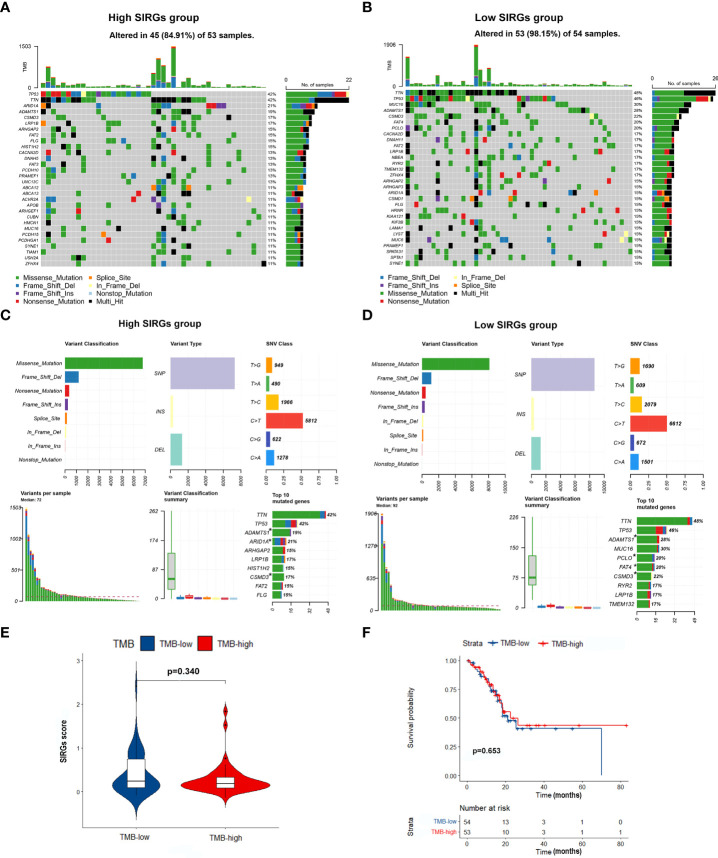
The mutation profile and TMB in low- and high-SIRGs score group. **(A)** Mutation profile of EGJA patients in high SIRGs score groups; **(B)** Mutation profile of EGJA patients in low SIRGs score groups; **(C)** The summary of mutation in high SIRGs score groups; **(D)** The summary of mutation in low SIRGs score groups; **(E)** The distribution of TMB in low- and high-SIRGs score group; **(F)** The association of TMB and OS.

As EGJA is a disease that features highly somatic alterations, we further detected the relationship between the SIRGs score and the TMB. The top 30 mutated genes in the high and low SIRGs score groups are shown in **Figures 6A, B**. We found that the mutations of ARID1A, ADAMTS1 and CSMD3 were high in the high SIRGs score group and rarely demonstrated in the low SIRGs group (**Figures 6C, D**). Moreover, the distribution of the SIRGs score was balanced in the high- and low-TMB groups (**Figure 6E**). Similarly, the OS showed no differences in the TMB groups (**Figure 6F**).

### The SIRGs score could be a predictive biomarker for immunotherapy

**Figure 7:**
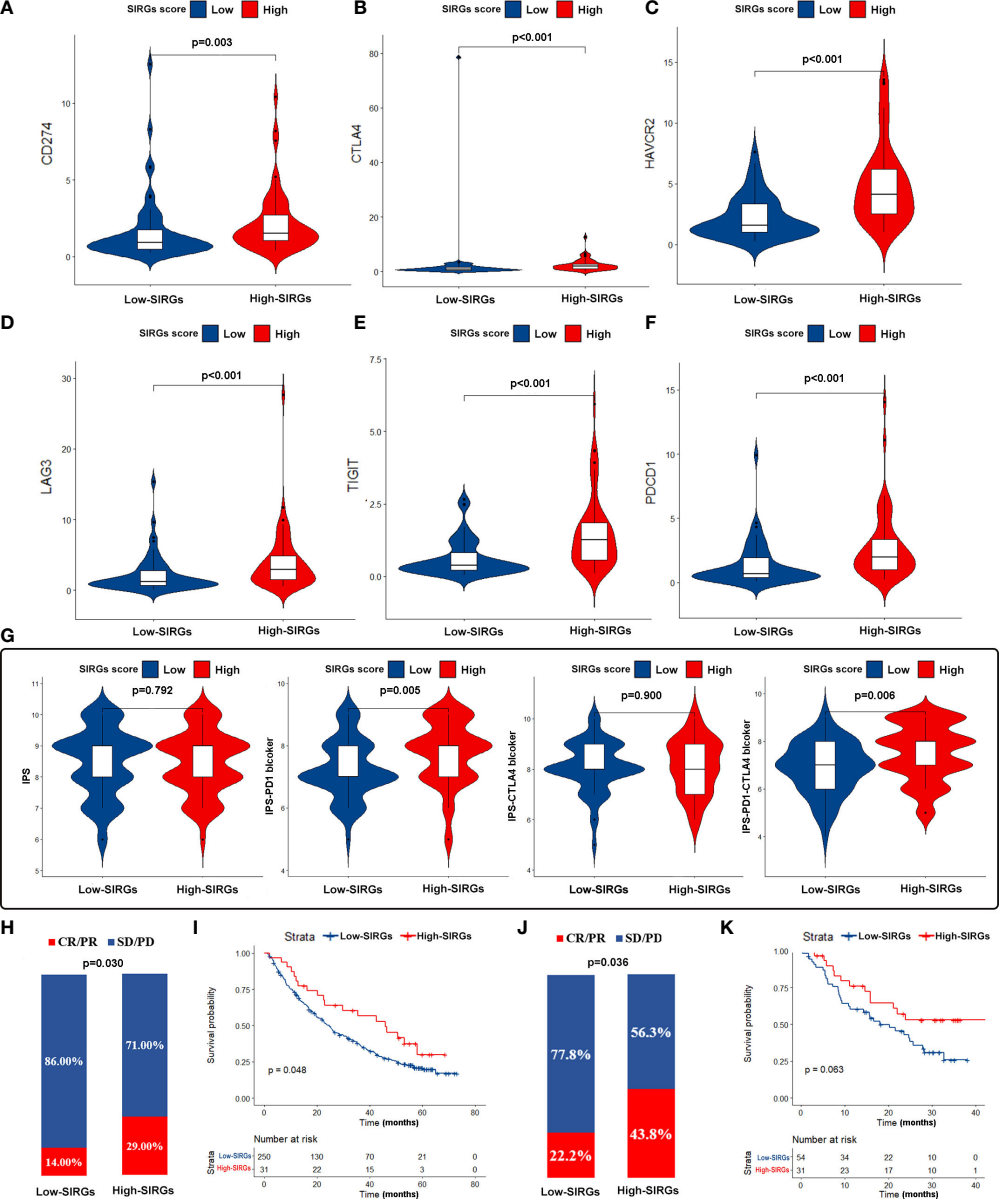
The estimation and validation of two SIRGs score groups in immunotherapy response. **(A-F)** The different expression of six immune checkpoint molecules (CD274, CTLA4, HAVCR2, LAG3, TIGIT, PDCD1) in different SIRGs score groups; **(G)** The association between IPS and SIRGs score; **(H)** The different immunotherapy response between two SIRGs score groups in advanced clear cell renal cell carcinoma cohort; **(I)** The association between SIRGs score and OS in advanced clear cell renal cell carcinoma cohort; **(J)** The different immunotherapy response between two SIRGs score groups in melanoma cohort; **(K)** The association between SIRGs score and OS in melanoma a cohort.

Our next step was to test whether the SIRGs score can be used as a biological target to predict the effectiveness of immunotherapy. We first examined the expression of immune checkpoints. The results showed that the expression of PDL1, CTLA4, HAVCR2 LAG3, TIGIT, and PD1 in the high SIRGs score group was increased significantly (**Figures 7A–F**). The IPS plays an essential role in evaluating the response to immune checkpoint inhibitors (ICIs) therapy. The IPS-PD1 and IPS-PD1-CTLA4 scores were higher in the high SIRGs score group (**Figure 7G**). Therefore, the above results indicate that EGJA patients with high SIRGs score may be more sensitive to immunotherapy. More importantly, we included 281 advanced clear cell renal cell carcinoma patients and 85 melanoma patients receiving immunotherapy for validation. In advanced clear cell renal cell carcinoma validation cohort, 29.0% of patients achieved complete response (CR) or partial response (PR) in the high SIRGs group (**Figure 7H**, p=0.030), which significantly improved the high SIRGs score patients’ OS (**Figure 7I**, P=0.048). In other words, patients in the low SIRGs group were not sensitive to immunotherapy, with only 14% CR/PR patients. Therefore, in the immunotherapy cohort, the low SIRGs patients’ prognosis was worse. Similarly, in melanoma cohorts (GSE78220 and GSE91061), 43.8% of patients with high SIRGs score reached CR/PR, with only 22.2% CR/PR patients in low SIRGs group (p=0.036, **Figure 7J**). The Kaplan–Meier survival analysis illustrated that there was a trend of better OS in high SIRGs group (p=0.063, **Figure 7K**).

## Discussion

EGJA is a malignant tumor in a special location. Different countries and regions have different treatment principles. Some studies in Europe and the United States have combined EGJA with esophageal cancer for research (31). While in Asia, clinical trials mostly combine EGJA and gastric cancer (32). Therefore, the biological characteristics of EGJA need to be further studied. However, immunotherapy has shown promising results in both esophageal cancer and gastric cancer (5). However, not all EGJA patients can benefit from immunotherapy. At present, there is still no good indicator to evaluate whether patients can benefit from immunotherapy before treatment, especially patients with EGJA. Studies have shown that the prognosis of gastric cancer patients and the effect of immunotherapy are related to the tumor immune microenvironment (13, 33). The stromal and immune cells crosstalk with cancer cells in tumor microenvironment. In the past, few researches fully considered the overall landscape of the infiltrating stromal and immune cells at the same time in tumor microenvironment. Therefore, we hope to construct a signature through stromal-immune related genes to predict the survival and immunotherapeutic effect of EGJA patients.

The ESTIMATE score is used to infer the infiltration of immune cells and stromal cells in the microenvironment of solid tumor tissues through the transcriptome data of tumor samples (17). Therefore, the SIRGs determined by ESTIMATE may be an important factor affecting the immune microenvironment. After calculating the stromal score and immune score of EGJA patients from TCGA, we identified 618 SIRGs. The results of KEGG and GO enrichment analyses suggested that the enriched pathways and functions are related to immunity, implying that the imbalance of these genes may cause changes in the immune microenvironment. Subsequently, we selected 122 SIRGs closely related to prognosis for LASSO regression and obtained the following 8 core genes: CFP, ZDHHC11, ASB5, LILRA4, FRZB, PTGDR, LRRC55 and FCN1. Then, we constructed the signature named SIRGs score through Cox regression analysis. Among them, CFP is a tumor prognostic marker associated with immune infiltration in gastric and lung cancer (34). ZDHHC11 can regulate the innate immune response to DNA viruses (35).

Further survival analysis confirmed that the SIRGs score can effectively predict the prognosis of this group of EGJA patients as an independent prognostic factor. As some studies have reported, the prognosis of EGJA was similar to that of GC, so to evaluate the postoperative prognosis of EGJA, they should be considered a part of GC instead of esophageal cancer (EC) (36, 37). In order to acquire sufficient cases to validate the SIRGs in predicting prognosis, we selected 733 GC patients as validation cohort. The results suggested that this score was also verified in gastric cancer data from GEO. Therefore, we can more effectively predict the prognosis of EGJA patients by TNM staging combined with the SIRGs score. These results suggest that this SIRGs score may be closely related to the biological behavior of the tumor itself and plays a unique role by changing the composition of the tumor microenvironment.

To further evaluate the relationship between the SIRGs score and the immune microenvironment, we used CIBERSORT to evaluate the infiltration abundance of 22 immune cells in the immune microenvironment of EGJA patients. Then, we divided them into four subtypes by unsupervised clustering. In theory, patients with more infiltrated and activated immune cells in the TME may have better immunotherapeutic effects (38). In subtype D, the infiltration of CD8+ T cells was more obvious than that of the other three subtypes, and activated CD4+ T cells and NK cells were also significantly increased. The immune and stromal scores were also higher in type D, suggesting that immune therapy may be more sensitive. In contrast, type C has fewer infiltrated CD8+ T cells and other immune cells and lower immune and stromal scores, which often indicates that the effect of immunotherapy is worse. Further analysis found that there were significant differences between high SIRGs score and low SIRGs score patients in the distribution of types C and D. High SIRGs score patients were mainly concentrated in subtype D, while low SIRGs score patients were mainly concentrated in subtype C. In addition, ssGSEA of immune-related functions showed that almost all immune-related functions in the high SIRGs score group were more active than those in the low SIRGs score group. Given the above, high SIRGs score patients with a “hot” immune microenvironment tend to have a relatively higher response rate to immunotherapy. However, we also found that the prognosis of EGJA patients with more obvious immune infiltration and more active immune function was worse. We speculate that this is due to the immune escape of tumor cells. Tumor cells and the TME are interdependent and antagonistic (39). The immunosuppressive tumor microenvironment is defined as the immunosuppressive part of the TME. Immune cells in the TME can always recognize and remove tumor cells in time. Immune escape means that tumor cells can avoid the recognition and attack of the immune system through various mechanisms to continue to grow and proliferate in the body (40). The immunosuppressive microenvironment consists of various immunosuppressive cells, immunosuppressive cytokines and various immune checkpoint molecules, which play an important role in tumor cell immune escape (41). As **Figure 5D** shows, gamma delta T cells, as a kind of immunosuppressive cell (42), were also upregulated in the TME of high SIRGs score patients. Current studies suggest that the upregulated expression of PD-L1 and CTLA4 on the surface of tumor cells plays a key role in the ability of tumor cells to escape from the host immune system. Therefore, we further compared the expression of immune checkpoint genes in tumor tissues of the high SIRGs score group and the low SIRGs score group and found that PD-L1, CTLA-4, HAVCR2, LAG3, TIGIT and PDCD1 were also up-regulated in the high SIRGs score group. Therefore, even if these patients had more obvious immune cell infiltration and the prognosis was still worse due to immune escape of the tumor, such patients might achieve better results after receiving immune checkpoint blockade treatment. Pornpimol Charoentong et al. used a random forest approach to identify determinants of immunogenicity and developed an immunophenoscore (IPS) based on the infiltration of immune subsets and the expression of immunomodulatory molecules (43). The IPS is a robust method for predicting anti-CTLA-4 and anti-PD-1 immunotherapy. It has been validated in independent cohorts. Furthermore, we investigated the relationship between IPS and different SIRGs score in EGJA patients. The results showed that the IPS-PD1 and IPS-PD1-CTLA4 scores were higher in the high SIRGs score group, indicating that they were more able to benefit from anti-PD-1 or anti-PD-1 plus anti-CTLA4 immunotherapy. The important role of PD-1/PD-L1 inhibitors in the therapy of some refractory tumors has been confirmed. However, in our study, IPS-PD1-CTLA4 scores also significantly improved in the high SIRGs group, suggesting that the SIRGs score may be able to identify EGJA patients who can benefit from PD-1/PD-L1 + CTLA4 inhibitor treatment. Checkmate 142, a phase II randomized controlled trial, demonstrated that nivolumab plus low-dose ipilimumab can significantly improve the disease control rate in metastatic colorectal cancer. However, the results regarding nivolumab in combination with ipilimumab in advanced GC or EGJA from Checkmate649 have not yet been published. The SIRGs score in our study may have predictive value to some extent.

To further confirm the efficacy of the SIRGs score, we selected two external cohorts of patients receiving immunotherapy for verification (44). In the advanced clear cell renal cell carcinoma cohort, 29% percent of the 31 patients in the high SIRGs group achieved complete response/partial response (CR/PR), while only 14% percent of 250 patients achieved CR/PR in the low SIRGs score group, and the p value of the chi-square test was 0.030. Kaplan–Meier survival curves revealed that the OS of high SIRGs score patients was better than that of low SIRGs score patients, with log-rank p=0.048, implying that immunotherapy may reverse the poor prognosis of high SIRGs score patients. The similar results were found in melanoma cohorts (GSE78220 and GSE91061). It was demonstrated that patients with high SIRGs score can significantly benefit from immunotherapy (p=0.036). Although the difference of Kaplan–Meier survival curves did not reach statistical significance (p=0.063), there was a trend towards better OS with immunotherapy in high SIRGs group. The deficiency may be attributed to insufficient sample in this cohort.

However, our research still has some limitations. First, we focused on one kind of malignant tumor at a specific site, so the overall number of cases and sequencing data are very limited. Second, the current obtainable cohort based on high-throughput sequencing to explore the efficacy of immunotherapy is very limited. We could only select another type of tumor for validation but not EGJA patients, and the number of cases in the validation group was also small. We should use EGJA cohort with immunotherapy for further verification in the future. Third, there is still a lack of some basic experiments to further explore the roles of these eight genes in changing the tumor microenvironment, which needs further research and exploration. Finally, in order to identify the cut-off value of SIRGs for distinguishing between high and low SIRGs group patients, we need include a large number of EGJA patients with immunotherapy for analysis in the future.

## Conclusion

In conclusion, the SIRGs score we constructed can effectively predict the prognosis of EGJA patients and prompt the tumor microenvironment of patients, providing a predictive role for the use of immunotherapy.

## Data availability statement

Publicly available datasets were analyzed in this study. This data can be found here: https://www.ncbi.nlm.nih.gov/geo/ and https://portal.gdc.cancer.gov/. The accession number(s) can be found in the article/**Supplementary Material**.

## Author contributions

SC was responsible for the design of this study. L-YO, Z-JD, and Y-FY analyzed the data and wrote the manuscript. J-MF, X-JC, J-JL, and X-ZL collected and cleaned the data. LL provided some suggestions for modification. All authors contributed to the article and approved to submit the final manuscript.

## Funding

This study Supported by Guangzhou Science and Technology Project (grant number 201803010040), Fund of the Sixth Affiliated Hospital of Sun Yat-sen University (grant number P20200217202309876) and National Key Clinical Discipline.

## Acknowledgments

We sincerely appreciate for public databases, such as GEO and TCGA.

## Conflict of interest

The authors declare that the research was conducted in the absence of any commercial or financial relationships that could be construed as a potential conflict of interest.

## Publisher’s note

All claims expressed in this article are solely those of the authors and do not necessarily represent those of their affiliated organizations, or those of the publisher, the editors and the reviewers. Any product that may be evaluated in this article, or claim that may be made by its manufacturer, is not guaranteed or endorsed by the publisher.
