# Supplementary figures and images for: SIRGs score may be a predictor of prognosis and immunotherapy response for esophagogastric junction adenocarcinoma
Source: Front Immunol. 2022 Aug 16;13:977894. doi: 10.3389/fimmu.2022.977894 (PMC9424497; doi:10.3389/fimmu.2022.977894)

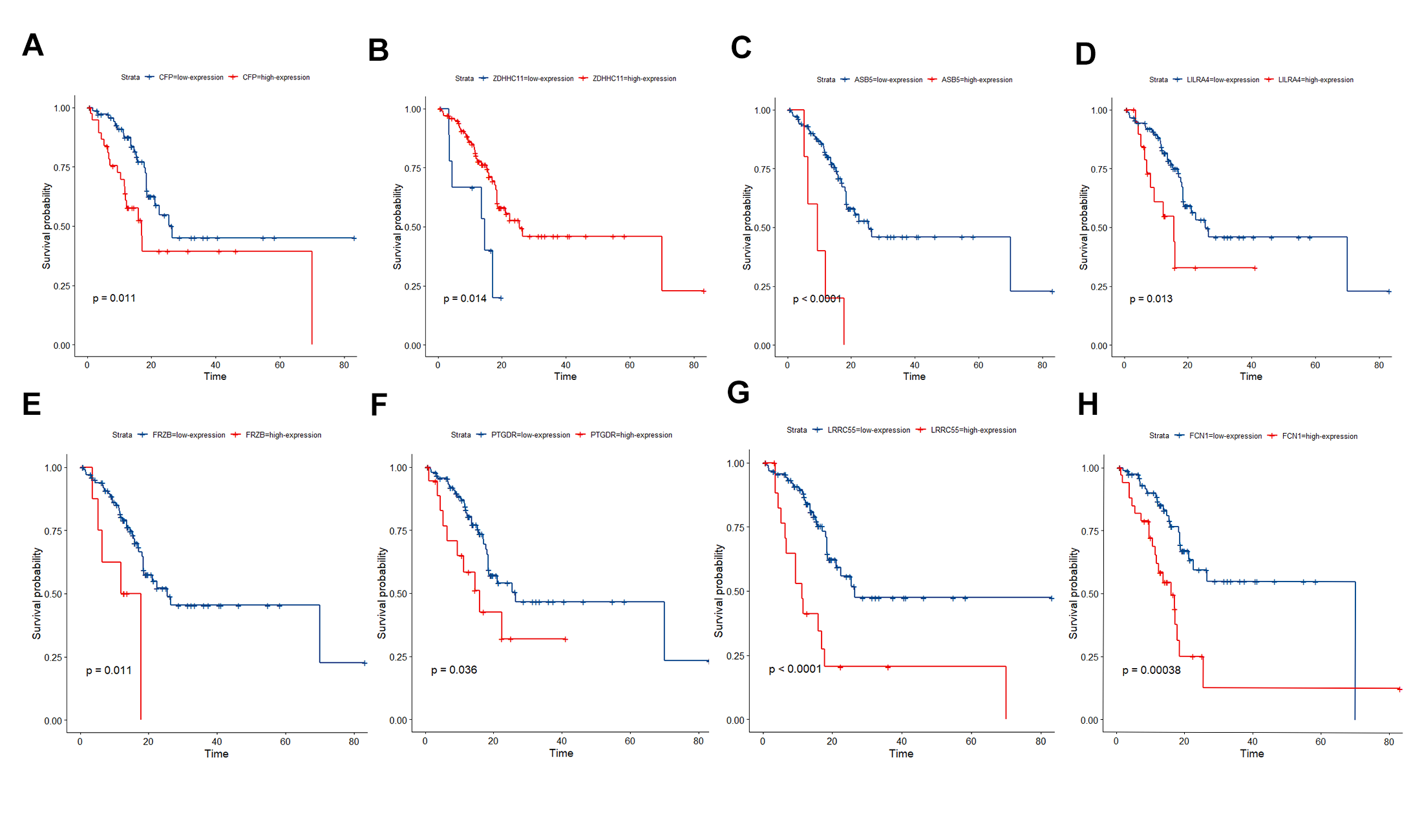

Supplement: Supplementary file 1 [file Image_1.tif]

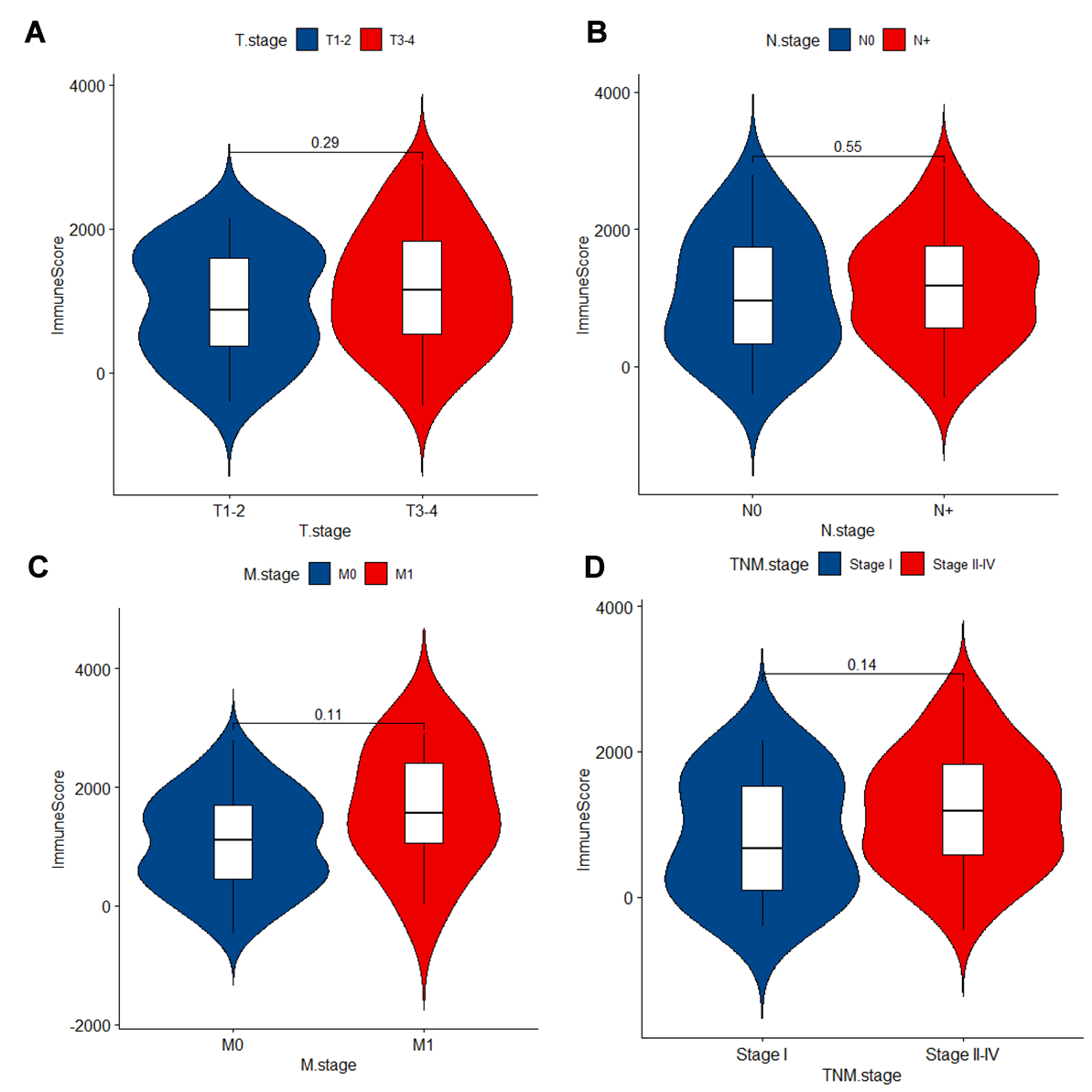

Supplement: Supplementary file 2 [file Image_2.tif]
